# Assessment of Fall Risk and Adverse Events Among General Ward Inpatients at a Regional General Hospital in Japan

**DOI:** 10.7759/cureus.77456

**Published:** 2025-01-15

**Authors:** Tetsuo Hagino, Tetsuhiro Hagino, Masanori Wako, Satoshi Ochiai, Naofumi Taniguchi, Takashi Ando, Jiro Ichikawa, Hirotaka Haro

**Affiliations:** 1 Department of Orthopaedic Surgery, National Hospital Organization (NHO) Kofu National Hospital, Kofu, JPN; 2 Department of Orthopaedic Surgery, University of Yamanashi, Chuo, JPN; 3 Department of Rehabilitation Medicine, Koshu Rehabilitation Clinics, Fuefuki, JPN

**Keywords:** adverse events, elderly patients, falls, inpatients, patient safety, risk factors

## Abstract

Introduction: Falls among hospitalized patients pose a significant concern for patient safety, influencing quality of life, mortality, and healthcare costs. This study aimed to assess the incidence of falls and related adverse events among general ward inpatients at a regional general hospital in Japan and identify associated risk factors.

Methods: The study involved 14650 patients aged 15 years or older, admitted between April 2016 and March 2023. Fall risk was evaluated using a 16-item assessment score sheet that considered factors such as age, history of falls, cognitive function, and physical condition. Patients were categorized into risk levels I, II, and III. Adverse events (level 3b or higher) were analyzed, and multivariate analysis was conducted to identify risk factors.

Results: Falls occurred in 373 patients (2.5%) during hospitalization, with a total of 475 falls and a fall rate of 1.99 per 1000 patient-days. Recurrent falls were noted in 67 patients. Higher fall risk was found in those aged 65 or older. Adverse events, including head injuries or fractures, affected 11 patients, mainly those aged 80 or older, with five undergoing surgery for hip fractures. Seven significant risk factors for falls were identified, including age ≥70 (odds ratio: 3.26), prior fall history (odds ratio: 1.58), and unstable gait (odds ratio: 2.07). The main risk factor for severe adverse events was needing assistance with mobility or excretion (odds ratio: 30.64).

Conclusions: This study identified the risk factors for falls and for adverse events in inpatients and demonstrated the need to pay special attention to patients who require assistance with mobility or excretion. These results suggest the importance of individualized interventions for fall prevention.

## Introduction

Falls on the same level and falls from a height (hereafter referred to as falls) among hospitalized patients have long been recognized as an important patient safety issue in clinical settings. Falls in hospitalized patients are a common medical accident, and approximately 2% of inpatients experience at least one fall during their hospital stay [1]. Hence, the impact of falls on patients' clinical outcomes cannot be neglected. About 30-50% of falls result in some kind of physical injury, and 1-3% cause fractures [2]. Orthopedic injuries and head injuries caused by falls may markedly reduce patients' quality of life and may even lead to increased mortality, prolonged hospital stay, and higher medical costs [3,4].

Japan has become a super-aging society, and the proportion of elderly patients among inpatients is increasing accordingly. Falls may become an even more serious issue in elderly patients and patients with comorbidities. Major orthopedic injuries such as hip fractures not only significantly elevate the mortality rate but also increase the risk of entering a nursing home after discharge [5]. Furthermore, the one-year mortality rate for hip fractures caused by in-hospital falls reaches 47%, which is markedly higher than the rate of 26% for hip fractures occurring in the community [5].

This study investigated the status of falls and adverse events among patients hospitalized in the general ward of a single regional general hospital in Japan, with the purpose of elucidating the factors impacting the occurrence of falls and the risk factors associated with adverse events.

## Materials and methods

The study was conducted at Kofu National Hospital, Kofu, Japan, after obtaining approval from the institution's Ethics Committee (approval number: R3-1). It involved 14650 patients aged 15 years or older, admitted between April 2016 and March 2023.

**Figure 1:**
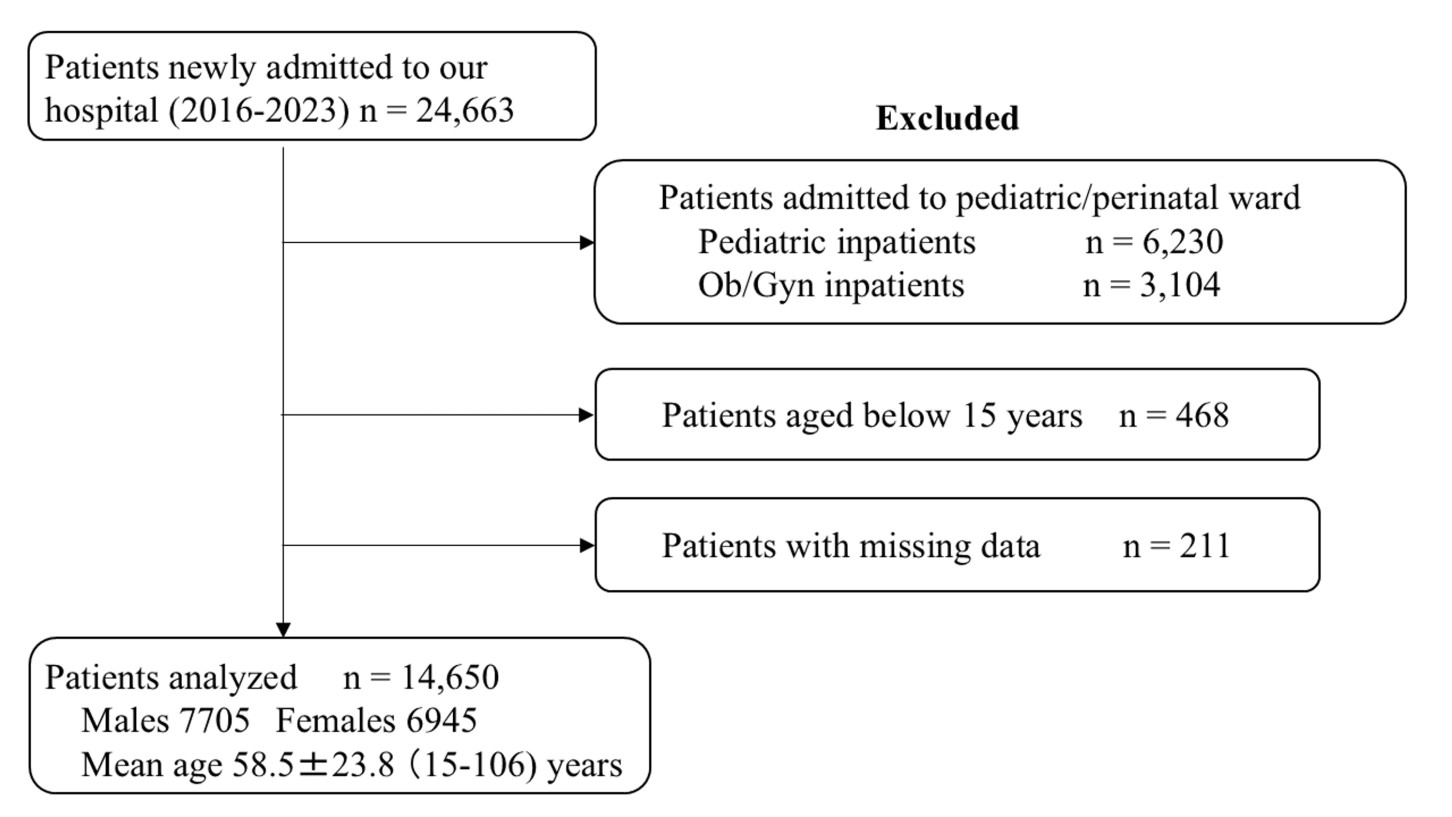
Flowchart of subject selection process

Our hospital is a regional core hospital with a total of 276 beds, comprising a ward for patients with severe motor and intellectual disabilities, a pediatric and perinatal ward, and an acute care general ward. Our hospital is unique in that most of the 100 beds in the general ward are occupied by orthopedic patients. The number of newly admitted patients over the eight years from April 2016 to March 2023 was 24663. After excluding 6230 pediatric patients and 3104 obstetric/gynecologic patients admitted to the pediatric and perinatal ward, 468 patients aged 15 years or below, and 211 patients with missing data, a total of 14650 patients were included as subjects for analysis (Figure 1).

The 14650 patients consisted of 7705 males and 6945 females, with a mean age of 58.5±23.8 (15-106) years. By clinical specialties, 10020 patients (68.4%) were in orthopedics, 2697 (18.4%) in gastrointestinal surgery, 1044 (7.1%) in general medicine, 873 (6%) in ophthalmology, and 16 (0.1%) in others.

Patient background data and results of fall risk assessment obtained at admission using the adult fall assessment score sheet were registered. Using the fall assessment score sheet (Table 1), patients were scored for 16 items regarding age, history of fall, environmental change, personality, physical function, cognitive function, activity status, and drug use. Based on the total scores obtained, the risk level was classified into I, II, and III. This sheet was developed by modifying the fall assessment sheet in the National Hospital Organization Fall Prevention Manual, and the validity of the sheet has been verified and reported [6]. The patient impact indicators used in this study were the National Hospital Organization Uniform Patient Impact Indicators (National Hospital Organization Guidelines for Medical Safety Management) (Table 2).

**Table 1:** Fall assessment score sheet Risk level I: scores 0-3; risk level II: scores 4-9; risk level III: scores 10 or above ADL: activities of daily living

Fall assessment score				At admission
Patient characteristics	Age		70 years or above	
	Past history		Had fall during the previous hospitalization or within the last three months	
	Environmental change	1	Is in a period of rapid recovery or worsening of the condition or ADL (including surgery)	
		2	Less than 7 days after admission or change of ward or room (for those aged 65 or above)	
		3	Is in a period of starting rehabilitation or undergoing training	
	Personality		Tends to act without pressing the nurse call button	
Patient condition	Physical condition	1	Leg strength and muscles are weakened	
		2	Can walk independently, but gait is unstable	
		3	Unstable when standing without support	
		4	Is bedridden, but can move the body in bed	
	Cognitive function		Memory and judgment are impaired	
	Activity state	1	Is using a wheelchair, walker, or handrail	
		2	Needs assistance with mobility and excretion	
	Drug use	1	Is using sleeping medication	
		2	Is using an anti-parkinsonian drug or muscle relaxant	
		3	Is using narcotic	
			Number of items checked (score 1 point for 1 item)/risk level	／

**Table 2:** Patient impact level

Level of incident	Outcome/treatment of injury
Level 1	Although an erroneous action was committed, it did not result in any impact on the patient
Level 2	The medical care or management provided caused an impact on the patient or possibly caused an impact on the patient
Level 3a	The medical care or management provided resulted in the need for minor treatment or procedure that was not otherwise required
Level 3b	The medical care or management provided resulted in the need for substantial treatment or procedures that were not otherwise required
Level 4	The medical care or management provided possibly caused serious permanent impairment that affects daily living
Level 5	The medical care or management provided caused death

In this study, level 3b (the medical care or management provided resulted in the need for substantial treatment or procedures that were not otherwise required) or higher in the classification was defined as the occurrence of an adverse event.

In this study, we investigated the occurrence of in-hospital falls and the impact of falls on patients. The occurrence of falls and the details were collected from all the incident reports submitted by nurses and hospital staff. Based on the above data, the relationship of the risk assessment results with the occurrence of falls and the level of impact was analyzed. Multivariate analysis was performed using the presence or absence of falls as the dependent variable and the 16 items in the fall assessment score sheet as independent variables. Furthermore, multivariate analysis was used to identify risk factors for the occurrence of adverse events.

For statistical analysis, univariate analyses were performed using the χ2 test and the Mann-Whitney U test. For multivariate analysis, covariates were entered by stepwise regression. A logistic regression model was fitted to assess the independent predictors. In all analyses, the significance level was set at less than 5%. Statistical analyses were performed using StatFlex Version 7 (Artec, Osaka, Japan).

## Results

**Figure 2:**
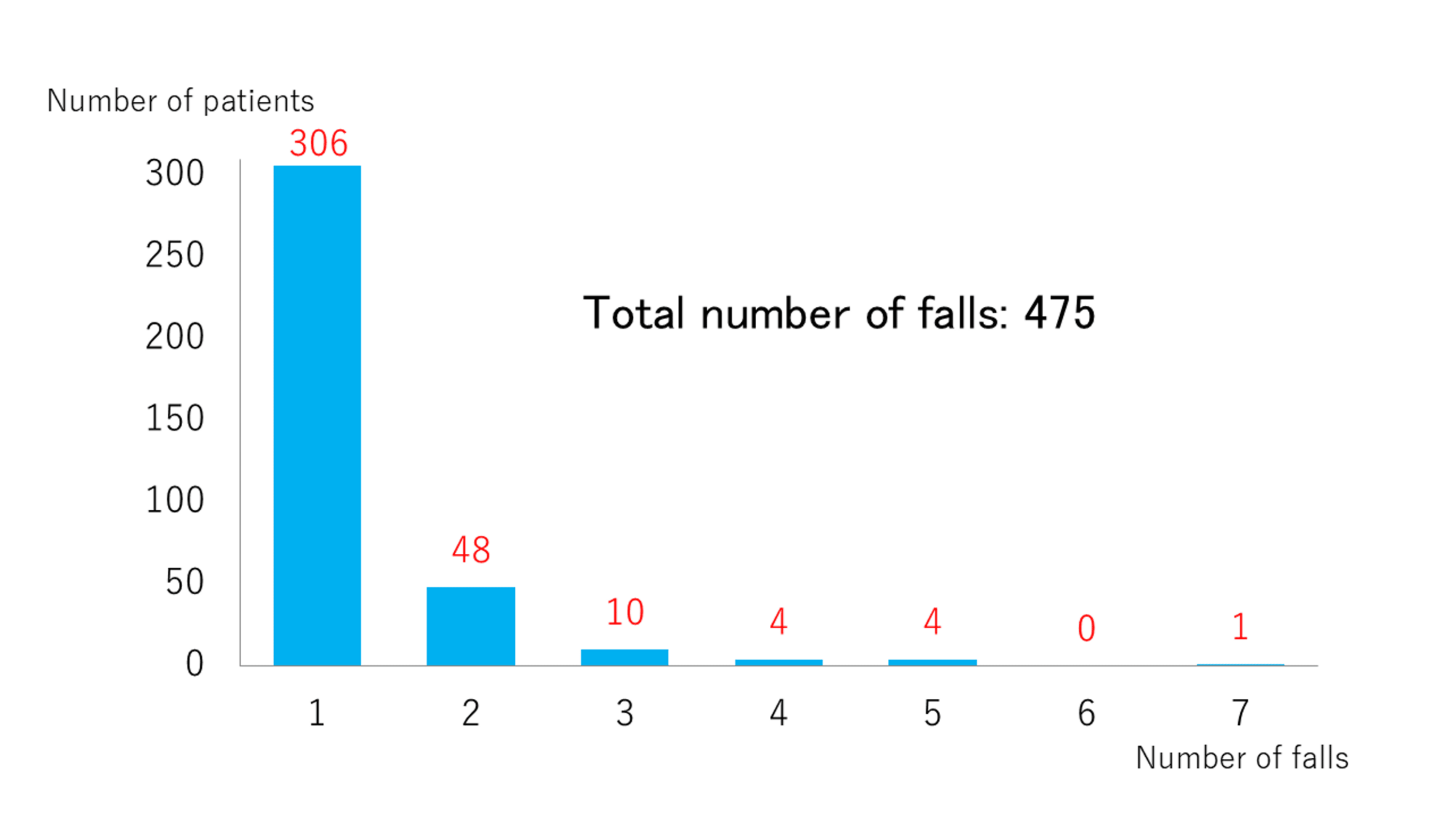
Number of falls among patients who experienced falls in the hospital Among 373 patients who had falls, 306 had only one fall, while 67 had repeated falls. Of a total of 475 falls, four patients had four falls, four had five falls, and one had seven falls

Falls occurred during hospitalization in 373 of 14650 patients (2.5%), with a total of 475 falls. Of the 373 patients, 306 experienced only one fall, 48 had two falls, 10 had three falls, four had four falls, four had five falls, and one had seven falls, with 67 patients having repeated falls (Figure 2). 

**Figure 3:**
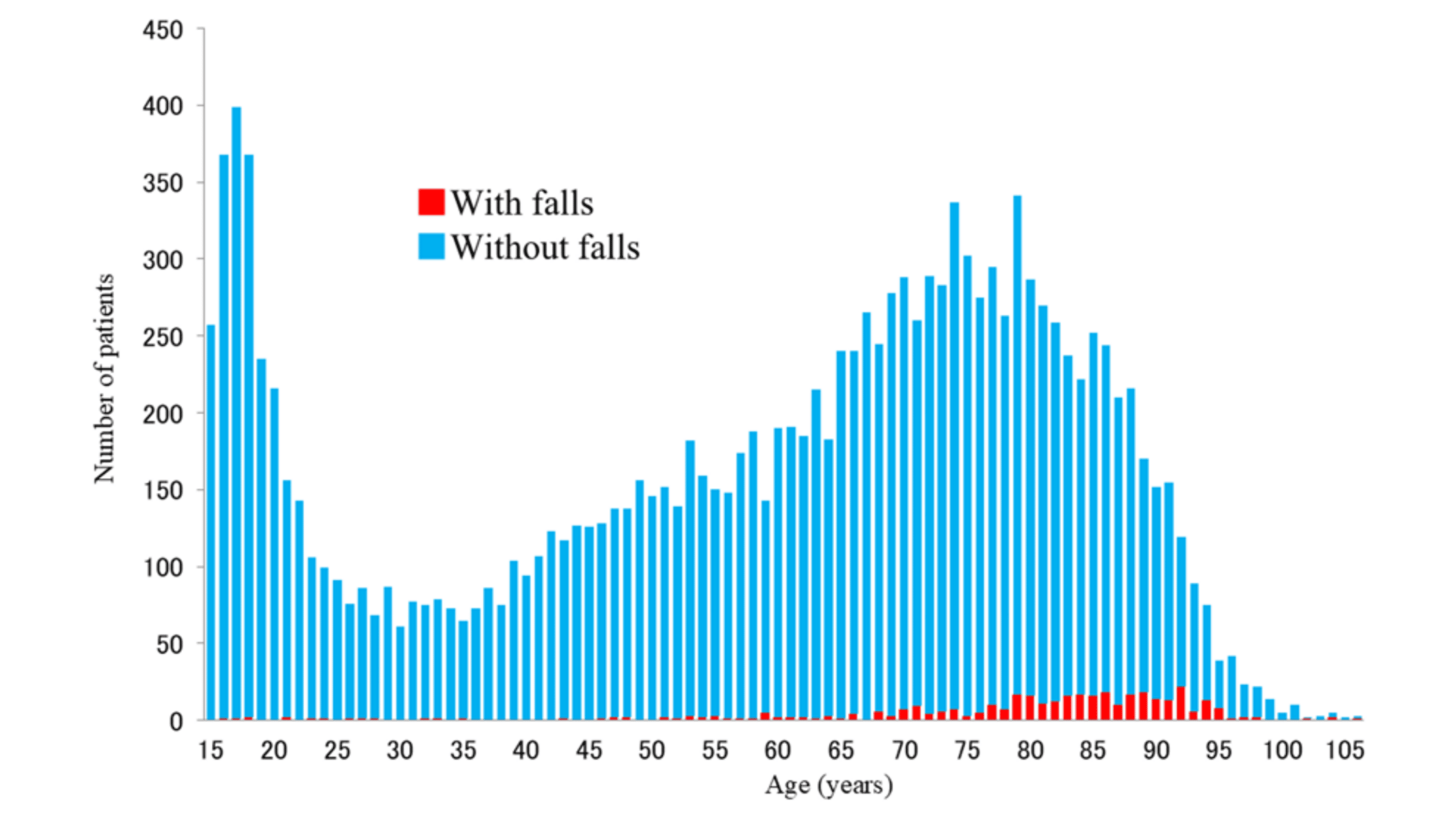
Age distribution of patients with and those without falls The red bars were those who fell and the mean age was 79.1±15.5 years, while the blue bars indicate a mean age of 57.9±23.7 years for those without falls, a significant difference (p<0.0001)

The fall rate (falls per 1000 patient-days) was 1.99. In the analysis of age distribution, falls occurred mostly in elderly patients aged 65 years or older. The mean age was 79.1±15.5 years in patients with falls compared with 57.9±23.7 years in those who did not fall and was significantly higher in patients with falls (p<0.0001) (Figure 3). 

Table 3 shows the number (rate) of patients with falls by risk level. Ninety-six patients (0.9%) with falls were classified as risk level 1, 241 patients (6.3%) as risk level II, and 36 patients (19.4%) as risk level III. The incidence of falls increased significantly with increasing risk levels (p<0.0001 by the chi-squared test) (Table 2). 

**Table 3:** Number of falls (fall rate) by risk level The incidence of falls increased significantly with increasing risk levels (p<0.0001 by the chi-squared test)

Risk level	Score	With falls (n=373)	Without falls (n=14277)	Total
Ⅰ	0-3	96 (0.9%)	10536 (99.1%)	10632
Ⅱ	4-9	241 (6.3%)	3591 (93.7%)	3832
Ⅲ	10 or above	36 (19.4%)	150 (80.6%)	186

Table 4 shows the relationship between risk level and patient impact level in patients with falls. 

**Table 4:** Relationship between risk level and patient impact level in patients with falls* *: number of patients (%) There was no relationship between risk level and patient impact level (p=0.056 by the chi-squared test)

	Level of incident	Risk level			
		Ⅰ	Ⅱ	Ⅲ	Total
Patient impact	Level 1	16 (16.7)	20 (8.3)	3 (8.3)	39
	Level 2	76 (79.2)	191 (79.3)	27 (75)	294
	Level 3a	4 (4.2)	20 (8.3)	5 (13.9)	29
	Level 3b	0 (0)	10 (4.1)	1 (2.8)	11
	Total	96	241	36	373

Thirty-nine patients with falls were classified in patient impact level 1, 294 patients in level 2, 29 patients in level 3a, and 11 patients in level 3b. Of the 11 patients in patient impact level 3b, 10 were at risk level II, showing no relationship between risk level and patient impact level. Among the 11 patients with patient impact level 3b, nine were aged 80 years or older, and all sustained head injuries or fractures; five patients underwent surgery, and one was transported as an emergency to a hospital specializing in neurosurgery (Table 5). 

**Table 5:** Individual cases with patient impact level 3b

Case	Age (years)	Sex	Specialty	Disease name at admission	Risk level	Injury due to fall	Surgery
1	90	Male	Orthopedics	Right femoral neck fracture	II	Subdural hematoma	None
2	94	Male	Surgery	Ascending colon cancer	II	Left femoral neck fracture	Bipolar hip arthroplasty
3	87	Female	Internal medicine	Lacunar infarction	II	Left trochanteric femoral fracture	Open surgery for fracture
4	92	Male	Orthopedics	Second lumbar vertebra compression fracture	III	Clavicle fracture, head contusion	None
5	84	Female	Orthopedics	Left elbow septic arthritis	II	Left trochanteric femoral fracture	None
6	83	Female	Orthopedics	Lumbar vertebral body fracture	II	Right femoral neck fracture	Open surgery for fracture
7	68	Female	Orthopedics	Right pubic bone fracture	II	Lumbar compression fracture	None
8	88	Male	Orthopedics	Left distal femoral fracture	II	Fracture of the skull, subdural hematoma	None, emergency transfer
9	75	Female	Orthopedics	Left distal femoral fracture	II	Peri-implant fracture	Open surgery for fracture
10	86	Male	Orthopedics	Right calcaneus fracture	II	Left trochanteric femoral fracture	Open surgery for fracture
11	95	Male	Internal medicine	Bradycardia arrhythmia	II	Ischium fracture	None

Multivariate analysis using presence of falls as the dependent variable and all 16 assessment items as independent variables identified seven items as risk factors for the occurrence of falls: age, past history of falls, tendency to act without pressing the nurse call button, unsteady gait, unstable when standing, impaired memory/judgment, and need for assistance with mobility/excretion (Table 6). 

**Table 6:** Assessment items identified as risk factors for the occurrence of falls Odds ratios were obtained by multiple stepwise regression (repeated forward selection) Presence of falls as the dependent variable and assessment items as independent variables

			Odds ratio	95% confidence interval	P-value
Patient characteristics	Age	70 years or older	3.26	2.40-4.41	<0.0001
	History	Has a past history of falls	1.58	1.23-2.03	0.0004
	Personality	Tends to act without pressing the nurse call button	1.48	1.07-2.06	0.0194
Patient conditions	Physical function	Can walk independently, but gait is unstable	2.07	1.59-2.71	<0.0001
		Unstable when standing without support	1.60	1.21-2.10	0.0009
	Cognitive function	Memory and judgment are impaired	1.75	1.32-2.33	0.0001
	Activity state	Needs assistance with mobility and excretion	1.70	1.29-2.25	0.0002

Furthermore, a multivariate analysis using the occurrence of adverse events (defined as a patient impact level of 3b or higher) as the dependent variable and all the assessment items as independent variables identified the need for assistance with mobility/excretion as the only factor significantly associated with the occurrence of adverse events. The odds ratio for this risk factor was 30.64, with a 95% confidence interval ranging from 3.72 to 252.43, and the result was statistically significant (p=0.0015).

## Discussion

The fall rate in acute care wards varies depending on the type of ward, patient characteristics, and disease and has been reported to range from 0.4 to 17 falls per 1000 patient-days [7-9]. In this study, the fall rate was 1.99, which is relatively low. This low rate may be attributed to the large proportion of young inpatients and various initiatives such as the development of nursing care plans for high-risk patients in our hospital.

The present study evaluated in detail the fall risk of inpatients at a Japanese regional general hospital and elucidated the risk of fall-related serious injuries, especially in elderly patients. We found that falls occurred in 373 patients, or 2.5% of the 14650 patients analyzed, and 67 of these patients experienced multiple falls. Notably, the risk of falls was elevated in elderly patients aged 65 years or older, and the mean age of patients who experienced falls (79.1 years) was significantly higher than those who did not fall (57.9 years).

Furthermore, serious injuries (patient impact level 3b) due to falls were concentrated in patients aged 80 years or older, revealing that elderly people are at high risk of sustaining serious complications such as fractures and head injuries from falls. Various risk factors for in-hospital falls have been reported, including psychiatric disease, alcohol abuse, dementia, delirium, male sex, a recent fall, gait instability, new urinary incontinence or frequency, adverse drug reactions (particularly associated with psychotropic drugs), and neuro-cardiovascular instability (most notably orthostatic hypotension) [2,10,11]. Johal et al. reported that inpatients who had hip fractures after falls tended to have many comorbidities and impaired cognitive function [5]. Furthermore, AlSumadi et al. analyzed 101183 acute admissions in a single UK district general hospital and reported that factors associated with moderate to severe harm included age and comorbidities, with 82% of patients being 75 years of age or older and 78% of patients having three or more comorbidities [12].

A previous study found that identifying multiple underlying risk factors and providing effective interventions to mitigate the impact of those risk factors reduced the incidence of inpatient falls by 20-30% [2]. In the present study, the assessment items that were identified to be risk factors for falls included a history of falls, physical function, and cognitive function, which are generally consistent with previous reports, reaffirming the need for interventions against these factors. On the other hand, the finding that the assessment item "needs assistance with mobility and excretion" was a risk factor for adverse events was a new discovery.

Approximately one-half of the 11 patients who sustained adverse events due to falls required surgery. This finding underscores the importance of fall prevention for hospitalized patients. However, based on the fall assessment score sheet evaluated at admission, the risk of falling in 10 of the 11 patients was level II, indicating the difficulty of predicting adverse events due to falls at the time of admission. However, multivariate analysis suggests that attention should be paid to patients who require assistance with mobility and excretion.

The limitations of the present study include a bias toward a large proportion of surgical patients, especially orthopedic patients, among the inpatients in our hospital; a relatively small number of adverse events; and data collection from a single facility, which limits the generalizability of the results. In addition, the assessment tool used in this study is affected by the subjective judgment of the assessor, and some items are difficult to judge objectively and accurately; there is room for improvement.

In the future, multi-facility and multi-regional research will be required to establish the risk factors for falls and develop effective preventive measures. In particular, further studies aiming at developing individualized fall prevention programs for elderly and orthopedic patients are needed. Evidence-based policies with the goal of reducing all falls may be the most effective approach to reduce the frequency of injuries and the associated costs. It is expected that multicenter collaborative research, the introduction of new fall prevention measures, and the verification of their effectiveness will contribute to safer medical care and reduced medical costs [13].

## Conclusions

This study evaluated fall risks and adverse events among inpatients in the general ward of a regional general hospital in Japan. The findings revealed that elderly patients, particularly those aged 80 years or older, are at a significantly higher risk of fall-related serious injuries, such as fractures and head injuries. Additionally, the need for assistance with mobility and excretion emerged as a strong predictor for adverse events, emphasizing the necessity for targeted interventions. These results underscore the importance of implementing individualized fall prevention measures and prioritizing high-risk patients to enhance patient safety and reduce healthcare costs.
